# Effect Modification of Hyperuricemia, Cardiovascular Risk, and Age on Chronic Kidney Disease in China: A Cross-Sectional Study Based on the China Health and Nutrition Survey Cohort

**DOI:** 10.3389/fcvm.2022.853917

**Published:** 2022-03-07

**Authors:** Yang Li, Bowen Zhu, Yeqing Xie, Shi Jin, Weiran Zhou, Yi Fang, Xiaoqiang Ding

**Affiliations:** ^1^Department of Nephrology, Zhongshan Hospital, Fudan University, Shanghai, China; ^2^Shanghai Medical Center of Kidney, Shanghai, China; ^3^Shanghai Key Laboratory of Kidney and Blood Purification, Shanghai, China

**Keywords:** hyperuricemia, cardiovascular disease risk, chronic kidney disease, interaction analysis, China health and nutrition survey

## Abstract

**Introduction:**

The question of whether the increased burden of chronic kidney disease (CKD) is caused by the interaction of hyperuricemia and cardiovascular disease (CVD) risk factors or is accelerated by aging remains unresolved. The purpose of this study is to better understand the effect modification of hyperuricemia, cardiovascular risk, and age on CKD among the Chinese population.

**Methods:**

This cross-sectional study of 8243 participants was derived from the China Health and Nutrition Survey (CHNS) in 2009. Inclusion criteria included age ≥18 years, non-pregnancy, and no history of high-protein diet prior to blood test. Demographics, comorbidities, health-related behaviors, and serum biomarkers were collected. Interaction association of hyperuricemia, CVD risk and age with CKD were analyzed using Logistic regression.

**Results:**

CKD was detected in 359 (27.2%, 95% CI 24.8∼29.7%) of 1321 participants with hyperuricemia and 680 (9.8%, 95% CI 9.1∼10.5%) of 6,922 participants without hyperuricemia, and these patterns remained significant after controlling for age, gender, and Framingham risk score (adjusted odds ratio [aOR] 3.82, 95% CI 3.20∼4.57). We found a negative multiplicative interaction between hyperuricemia and CVD risk on CKD. The aOR in low-CVD risk groups was 5.51 (95% CI 4.03∼7.52), followed by medium-CVD risk groups (aOR: 3.64, 95% CI 2.61∼5.09) and high-CVD risk groups (aOR: 2.89, 95% CI 2.12∼3.96). CVD risk was less associated with CKD in hyperuricemia group (aOR: 0.92, 95% CI 0.68∼1.22) than in non-hyperuricemia group (aOR: 1.43, 95% CI 1.21∼1.70). Furthermore, hyperuricemia and age had a significant additive effect on CKD, with a synergy index of 2.26 (95% CI 1.45∼3.52). Coexisting with older age and hyperuricemia, the likelihood of developing CKD was higher than the sum of the two alone.

**Conclusion:**

The link between hyperuricemia and CKD begins at a young age and becomes stronger in the low CVD risk group. For young adults, early detection of hyperuricemia, routine CVD risk assessment, and timely intervention of modifiable factors are warranted.

## Introduction

Chronic kidney disease (CKD) is a growing public health concern around the world (1). According to the Global Burden of Disease Study, there were 697⋅5 million CKD cases in 2017, which was considerably higher than those individuals with diabetes, asthma, osteoarthritis and chronic obstructive pulmonary disease (COPD) (2, 3). China has the most CKD patients (132.3 million), accounting for one-fifth of all cases worldwide. If left untreated, CKD can progress to end-stage renal disease (ESRD), which necessitates renal replacement therapy and hurts the quality of life (4). The global all-age mortality rate from CKD has increased 41⋅5% in the last three decades (2). Impaired kidney functions also contributed to 7⋅6% of cardiovascular disease (CVD) deaths (2). Therefore, the importance of CKD prevention and early detection should pose the same priority as the management of diabetes and hypertension.

The kidney is the major excretory and endocrine organ of the body and plays an important role in maintaining homeostasis. Cardiovascular function and metabolic status are closely related to kidney function (5–7). Previous evidence suggests that CVD risk factors (hypertension, diabetes, smoking, and alcohol drinking) and elevated serum uric acid are associated with an increased incidence of CKD (8, 9). Kidney dysfunction, in turn, accelerates the onset of CVD and impairs urate excretion (10, 11). However, the relative importance of CVD risk and hyperuricemia to CKD has not been well established. Furthermore, as an aging-related disease, CKD was more common in older adults (12). This complicates determining whether the increased CKD burden is due to the interaction of hyperuricemia and CVD risk or is accelerated by aging itself. Most of the previous studies were limited by insufficient sample size and a lack of age-stratified analysis to quantify the effect modification of hyperuricemia, CVD risk, and age on CKD.

We hypothesized that hyperuricemia and CVD risk factors had complex interactions with CKD and this process is modified by age as well. To this end, we conducted a cross-sectional analysis derived from the China Health and Nutrition Survey (CHNS) cohort to better understand the association and interaction effects of hyperuricemia, CVD risk, and age on CKD.

## Materials and Methods

### Study Design and Patients

**FIGURE 1:**
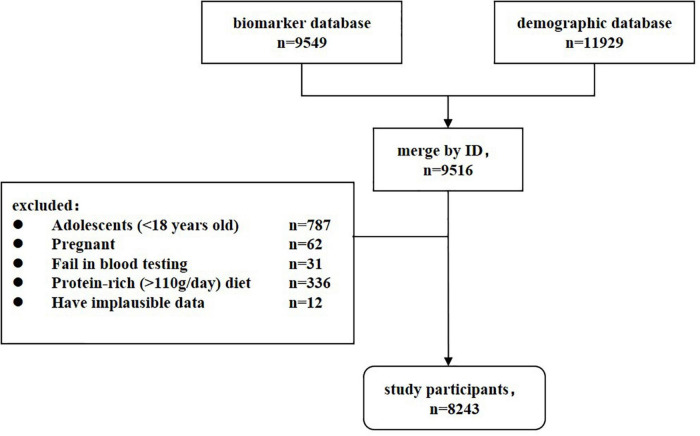
Flow diagram for selecting participants.

China health and nutrition survey is a China-wide prospective cohort study that is currently being conducted in fifteen provinces and autonomous cities/districts (Beijing, Chongqing, Guangxi, Guizhou, Heilongjiang, Henan, Hubei, Hunan, Jiangsu, Liaoning, Shaanxi, Shandong, Shanghai, Yunnan, and Zhejiang). For each study field, community-based participants were recruited through multistage, random cluster sampling. The survey began in 1989 and follow-up data collection has occurred every 2–4 years since then. CHNS has completed ten waves by 2015. An in-depth description of CHNS was can be found at https://www.cpc.unc.edu/projects/china/. In 2009, fasting blood was collected for the first time, covering twenty-six measures. Major CVD biomarkers (lipids, HbA1c, glucose, insulin, triglycerides), renal biomarkers (urea, creatinine), metabolic biomarkers (uric acid, cholesterol, and triglyceride), and important nutrition biomarkers (transferrin, hemoglobin, albumin, and ferritin) were included. Our study was designed to be cross-sectional. A total of 9,516 participants who provided blood samples and anthropometry data were recruited as the study population. After exclusion of 787 adolescents (<18 years old), 62 pregnant women, 31 individuals failing in blood test, 336 individuals on a high protein-rich diet (>110g/day) (13), and 12 individuals with implausible data, 8,243 participants were eligible for the formal analysis (Figure 1). When using the sample size formula for ($n=u_{\alpha /2}^{2}\,\pi \,\left(1-\pi \right)/\delta^{2}$) and setting the CKD prevalence (π) to 10.8% (12) and the limit error (δ) to 3%, a minimum of 411 subjects would have been needed to achieve 90% power and 5% significance level. The present sample size provides adequate statistical capacity. The institutional review boards of the University of North Carolina (United States) and the National Institute of Nutrition and Food Safety (China) approved the ethics of CHNS (14). All participants signed written informed consent forms and their identities were withheld from the public datasets.

### Data Collection and Measurements

Standardized questionnaires were used to collect demographic information (age, gender, education, residence, nationality and family income), comorbidity (hypertension, diabetes and myocardial infarction), and health-related behaviors (smoking, alcohol consumption and physical exercise). Age was grouped into 15-year age categories: 18∼29 years, 30∼44 years, 45∼59 years, 60∼74 years, and 75∼ years. Education was divided into never, primary school, junior high school, senior high school, and post-secondary education. Health-related behaviors included smoking status (never, former, or current smoker), alcohol use (never, former, or current drinker), and physical activity. We applied metabolic equivalents (MET) per week to quantify physical activity and classified it into three groups: <57.17 MET/week (low tertile T1), 57.17–148.06 MET/week (medium tertile T2), and ≥148.06 MET/week (high tertile T3) (15). Additionally, anthropomorphic and blood pressure (BP) measurements were taken from the participants. Body mass index (BMI) was calculated as weight in kilograms divided by height in meters squared. Waist-hip ratio was calculated by dividing the waist circumference (cm) by the hip circumference (cm). BP was measured at rest with a mercury sphygmomanometer and collected in triplicate to obtain the mean value. Hypertension was defined as a systolic BP ≥140 mmHg or diastolic BP ≥90 mmHg, or self-reported diagnosis by questionnaire. Diabetes was defined by self-reported or diabetes treatment records. About 12 ml of fasting blood was drawn by venipuncture. The serum was separated and stored in the ultra-low-temperature freezer. All samples were analyzed in Beijing at a national central lab under strict quality control (16). Serum uric acid was detected using enzymatic colorimetric method (Hitachi 7600 automated analyzer). Serum creatinine was detected using picric acid method. Total cholesterol (TC) was detected by CHOD-PAP method, and high-density lipoprotein cholesterol (HDL-C) was detected by enzymatic method. Hemoglobin A1c (HbA1c) was detected in high-performance liquid chromatography system (HLC-723 G7).

### Definition of Hyperuricemia, Cardiovascular Disease Risk, and Chronic Kidney Disease

Hyperuricemia was defined as a fasting serum uric acid level >420 μmol/L in males and >360 μmol/L in females, consistent with other studies (17, 18). CVD risk was assessed using the Framingham risk score (FRS), which can predict 10-year CVD risk based on factors including age, gender, TC, HDL-C, smoking status, BP, and diabetes (19, 20). CVD risk was graded as low (FRS <10%), medium (FRS 10–20%), or high (FRS >20%) (21). We applied the CKD-EPI 2009 creatinine equation to calculate the estimated glomerular filtration rate (eGFR) (22). The outcome variable of CKD was defined as eGFR <60 mL/min/1.73 m^2^ according to Kidney Disease: Improving Global Outcomes (KDIGO) 2012 guideline (4).

### Statistical Analysis

Continuous data were expressed as mean ± standard deviation or median (interquartile range), and categorical data were expressed as frequency (percentage). Group comparisons between continuous variables were assessed using Student’ *t*-test and Mann–Whitney test when appropriate. Pearson chi-square test was used for binary and unordered categorical variables, whereas Cochran-mantel-haenszel test was used for ordinal categorical variables. Logistic regression was done to analyze the association of hyperuricemia and CVD risk with CKD. We further introduced a multiplicative interaction term (hyperuricemia × CVD-risk × age) into the model to investigate effect modification. Subsequently, subgroup analyses were performed by adjusting for age, gender, and FRS (or uric acid) depending on which association was examined. In addition, we did analyses to examine the additive interaction of age+hyperuricemia and age+CVD-risk. The synergistic effect was quantified using relative excess risk due to interaction (RERI), attributable proportion due to interaction (AP), and synergy index (SI). Sensitivity analyses were done to assess the robustness of the results by treating eGFR as a continuous variable. When the 95% confidence interval (CI) for RERI, AP, and SI did not overlap 0, 0 and 1, respectively, significant additive interaction was observed. A two-tailed *p* < 0.05 was considered the statistical significance threshold. Analyses were performed using the package of “ggplot2,” “gmodels” and “epiR” in the R 3.6.1 software (R core team).

## Results

### Baseline Characteristics

In total, 8,243 participants were recruited in the final analysis (Figure 1). The mean age was 51.59 ± 15.28 years, and the gender ratio was 0.85 (male 3790/female 4453). Approximately 16.0% of participants had hyperuricemia and 12.6% had CKD. Men and well-educated participants had a higher prevalence of hyperuricemia, whereas women and participants with illiteracy or primary education had a higher prevalence of CKD (Table 1). Older age, urban residence, and higher family income were all associated with both hyperuricemia and CKD. Compared to healthy controls, participants with hyperuricemia and CKD had more CVD risk factors, including former smoking, hypertension, diabetes, overweight, and lower physical activities. These resulted in an overall higher FRS value (*p* < 0.001) and a greater proportion of medium- and high-CVD risk among participants (*p* < 0.001) with hyperuricemia and CKD. Notably, current smokers and drinkers were more susceptible to hyperuricemia but had the lowest prevalence of CKD. The sensitivity analysis between analytic participants and excluded participants found that the proportions of illiteracy, suffering from comorbidities and never smoker/drinkers were lower in the excluded group (Supplementary Table 1), which was primarily attributed to the exclusion criteria for adolescents (<18 years old).

**TABLE 1:** Baseline characteristics of the participants stratified by hyperuricemia and chronic kidney disease (*n* = 8243).

	Hyperuricemia		Statistics	*P*-value	Chronic kidney disease		Statistics	*P*-value
	Yes (*n* = 1321)	No (*n* = 6922)			Yes (*n* = 1039)	No (*n* = 7204)		
**Demographics**								
Age (years)	54.21 ± 15.55	51.09 ± 15.17	6.813	<0.001^a^	68.85 ± 11.02	49.10 ± 14.15	43.130	<0.001^a^
Male (%)	801(60.6)	2989(43.2)	136.067	<0.001^c^	411(39.6)	3379(46.9)	19.735	<0.001^c^
Education (years)			11.891	0.018^d^			410.065	<0.001^d^
Never	167(12.7)	898(13.0)			300(29.0)	765(10.6)		
Primary school	360(27.3)	2016(29.2)			395(38.1)	1981(27.5)		
Junior high school	425(32.3)	2364(34.2)			175(16.9)	2614(36.4)		
Senior high school	163(12.4)	786(11.4)			61(5.9)	888(12.3)		
Post-secondary	202(15.3)	846(12.2)			105(10.1)	943(13.1)		
education								
Urban residence (%)	522(39.5)	2208(31.9)	29.058	<0.001^c^	410(39.5)	2320(32.2)	21.587	<0.001^c^
Nationality (Han)	1154(87.8)	6120(88.7)	0.933	0.334^c^	896(86.6)	6378(88.8)	4.502	0.034^c^
Total net	12575 [7200,21702]	10800 [5331,18210]	6.760	<0.001^b^	12000 [6260,19800]	10882 [5487,18807]	2.295	0.022^b^
individual								
income (CNY)								
**Comorbidity**								
Hypertension	554(41.9)	1761(25.4)	149.481	<0.001^c^	572(55.1)	1743(24.2)	428.107	<0.001^c^
Diabetes	105(8.0)	309(4.5)	28.235	<0.001^c^	119(11.5)	295(4.1)	102.988	<0.001^c^
Myocardial infarction	32(2.4)	103(1.5)	6.092	0.014^c^	51(4.9)	84(1.2)	78.351	<0.001^c^
**Health-related behaviors**								
Smoking status			72.231	<0.001^c^			45.956	<0.001^c^
Never smoker	655(49.6)	4291(62.0)			634(61.0)	4312(59.9)		
Former smoker	227(17.2)	846(12.2)			192(18.5)	881(12.2)		
Current smoker	439(33.2)	1785(25.8)			213(20.5)	2011(27.9)		
Alcohol drinking			84.729	<0.001^c^			103.604	<0.001^c^
Never drinker	508(38.5)	3504(50.6)			531(51.1)	3481(48.3)		
Former drinker	258(19.5)	1348(19.5)			298(28.7)	1308(18.2)		
Current drinker	555(42.0)	2070(29.9)			210(20.2)	2415(33.5)		
Physical exercise level			22.488	<0.001^d^			310.029	<0.001^d^
Low	443(36.9)	2083(32.7)			505(57.5)	2021(30.2)		
Medium	427(35.6)	2090(32.8)			267(30.4)	2250(33.7)		
High	329(27.4)	2191(34.4)			106(12.1)	2414(36.1)		
**Anthropometry**								
WHR	0.90 ± 0.07	0.87 ± 0.08	12.916	<0.001^a^	0.89 ± 0.08	0.87 ± 0.08	7.611	<0.001^a^
BMI (kg/m^2^)	24.72 ± 3.64	23.08 ± 3.39	15.784	<0.001^a^	23.41 ± 3.81	23.33 ± 3.44	0.692	0.489^a^
Systolic BP (mm Hg)	130.02 ± 19.73	123.99 ± 18.96	9.772	<0.001^a^	137.27 ± 21.86	123.02 ± 18.01	22.135	<0.001^a^
Diastolic BP (mm Hg)	83.19 ± 11.73	79.63 ± 11.02	9.882	<0.001^a^	82.28 ± 11.80	79.88 ± 11.08	6.211	<0.001^a^
**Blood biomarkers**								
FRS score	10.0 [5.3,18.5]	5.6 [2.8,11.7]	−16.752	<0.001^b^	13.7 [8.6,28.5]	5.5 [2.8,11.7]	−26.878	<0.001^b^
CVD risk			206.676	<0.001^d^			624.034	<0.001^d^
Low	610(46.7)	4649(67.4)			317(31.2)	4942(68.8)		
Medium	388(29.7)	1296(18.8)			323(31.8)	1361(18.9)		
High	309(23.6)	951(13.8)			377(37.1)	883(12.3)		
eGFR (mL/min/1.73 m2)	70.74 ± 18.66	80.13 ± 16.10	−18.904	<0.001^a^	50.87 ± 8.57	82.63 ± 13.73	−72.537	<0.001^a^
SUA (μmol/L)	471.52 ± 120.98	276.56 ± 65.91	83.882	<0.001^a^	358.32 ± 97.49	300.52 ± 104.50	16.807	<0.001^a^

*^a^Student’s t-test; ^b^Mann–Whitney test; ^c^Pearson test; ^d^Cochran-mantel-haenszel test; CNY, China Yuan; WHR, waist to hip circumference ratio; BMI, body mass index; BP, blood pressure; FRS, Framingham risk score; eGFR, estimated glomerular filtration rate; CKD, chronic kidney disease; SUA, serum uric acid.*

*In total, 16 participants were not available for education level; 27 participants were not available for nationality; 2,193 participants were not available for total net individual income; 19 participants were not available for diabetes; 66 participants were not available for myocardial infarction; 680 participants were not available for physical exercise level; 234 participants were not available for WHR; 154 participants were not available for BMI; 1,120 participants were not available for SBP/DBP; and 51 participants were not available for FRS.*

### Age-Specific Association Analysis of Hyperuricemia and Cardiovascular Disease Risk With Chronic Kidney Disease

Chronic kidney disease (eGFR <60 mL/min/1.73 m^2^) was found in 359 (27.2%, 95% CI 24.8∼29.7%) of 1321 participants with hyperuricemia and 680 (9.8%, 95% CI 9.1∼10.5%) of 6922 participants without hyperuricemia. Patterns remained significant after adjusting for age, gender, and FRS (adjusted odds ratio [aOR] 3.82, 95% CI 3.20∼4.57). The likelihood of CKD increased along with the progression of CVD risk strata (6.0% vs. 19.2% vs. 29.9%, *p* < 0.001), and this positive significant association was maintained in the adjusted model (aOR 1.37, 95% CI 1.20∼1.56). Similarly, eGFR values were lower in participants with hyperuricemia and a high-CVD risk than healthy controls, with the differences of −8.04 (95% CI −8.79∼−7.30) and −1.33 (95% CI −1.85∼−0.81), respectively.

**FIGURE 2:**
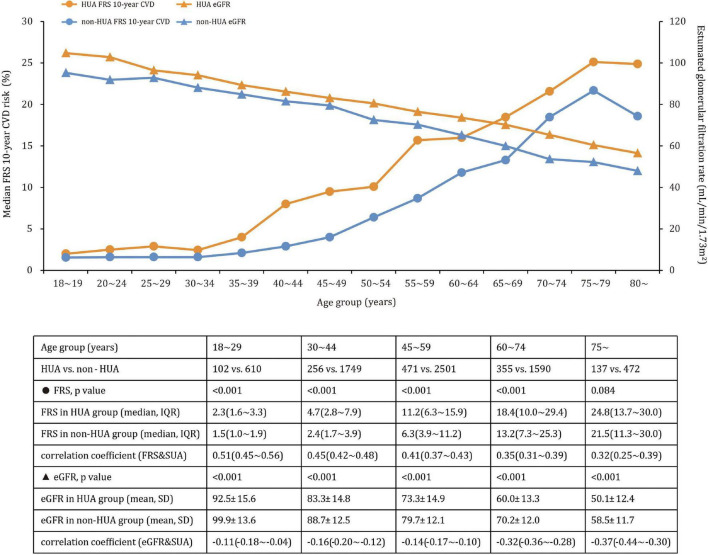
Mean eGFR (SD) and median CVD risk (interquartile range) across age groups in participants with and without hyperuricemia.

Participants with hyperuricemia had higher FRS values than participants without hyperuricemia in the 18∼29, 30∼44, 45∼59, and 60∼74 age groups, but the difference was no longer significant in the 75∼ age group (Figure 2). In contrast, significant higher proportions of CKD were observed in participants with hyperuricemia in age groups 30∼44 years (4.3% vs. 0.7% [*p* < 0.001]), 45∼59 years (15.9% vs. 4.6% [*p* < 0.001]), 60∼74 years (46.8% vs. 16.8% [*p* < 0.001]) and 75∼ years (77.4% vs. 54.0% [*p* < 0.001]), but not at 18∼29 years (1.0% vs. 0.2% [*p* = 0.266]), implying larger differences in older age. A comparison of eGFR estimates across age groups in participants with and without hyperuricemia revealed similar patterns, as did the correlation analysis among FRS, eGFR, and uric acid.

### Multiplicative Interaction Models of Hyperuricemia, Cardiovascular Disease Risk, and Age on Chronic Kidney Disease

**FIGURE 3:**
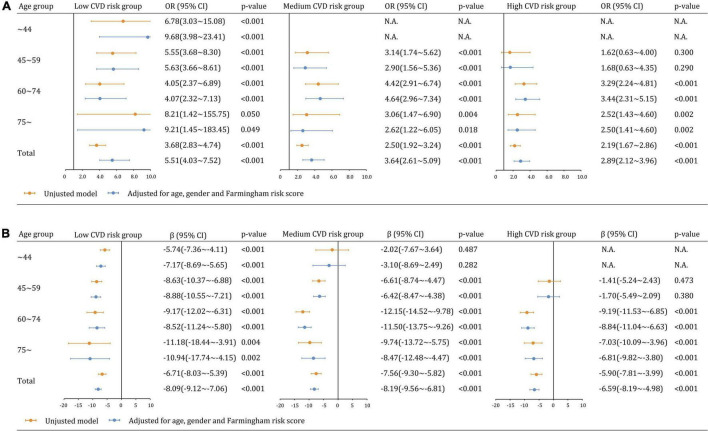
Multiplicative interaction models examining the relationships between hyperuricemia and kidney dysfunction across CVD risk and age groups **(A)** CKD (eGFR <60 mL/min/1.73 m^2^). **(B)** eGFR as a continuous variable.

A significant negative interaction between hyperuricemia and CVD risk was observed on the likelihood of CKD in the multivariable model (β_hyperuricemi*CVD–risk_ = −0.343, *p* = 0.001), showing that the effect of hyperuricemia on CKD attenuated with increased CVD risk (Figure 3A). In particular, hyperuricemia was associated with CKD, independent of age, gender and FRS, in low-CVD risk groups (aOR: 5.51, 95% CI 4.03∼7.52), medium-CVD risk groups (aOR: 3.64, 95% CI 2.61∼5.09), and high-CVD risk groups (aOR: 2.89, 95% CI 2.12∼3.96). The aOR curve was an inverted parabola in the medium- and high-CVD groups and participants aged 60∼74 years had the highest CKD prevalence (aOR 4.64 and 3.44 [all *p* < 0.001]). In a sensitivity analysis using eGFR as a continuous variable, the interaction between hyperuricemia and CVD risk was positively related to eGFR value (β_hyperuricemia*CVD risk_ = 1.89, *p* = 0.002). Furthermore, there was an interaction between hyperuricemia and age, negatively influencing eGFR (β_hyperuricemia*age_ = −1.57, *p* = 0.002 in Figure 3B).

**FIGURE 4:**
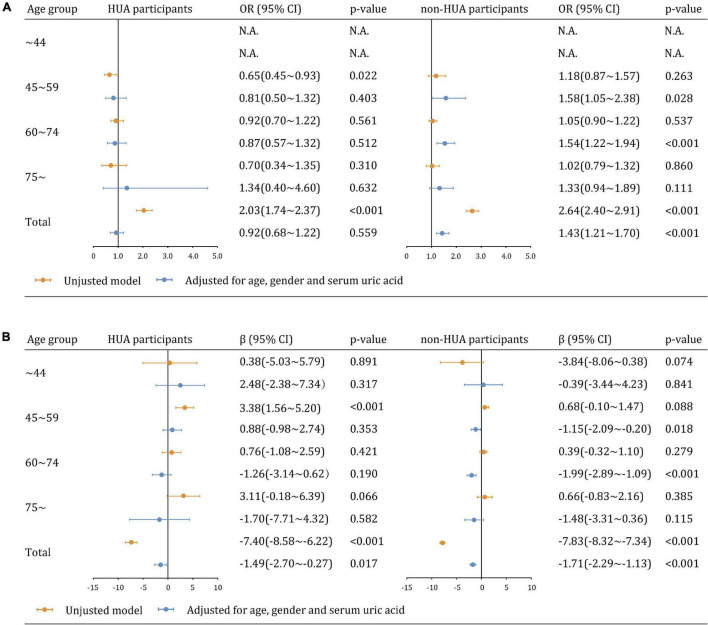
Multiplicative interaction models examining the relationships between CVD risk and kidney dysfunction across hyperuricemia and age groups **(A)** CKD (eGFR <60 mL/min/1.73 m^2^). **(B)** eGFR as a continuous variable.

We further analyzed the relationship between CVD risk and CKD across age groups. CVD risk was less likely to be significantly associated with CKD and eGFR estimates in participants with hyperuricemia than those without hyperuricemia (Figure 4A). The adjusted associations between CVD risk and CKD in participants with hyperuricemia were not significant overall (aOR: 0.92, 95% CI 0.68∼1.22 [*p* = 0.559]) and in all age groups. In contrast, the associations became significant among participants without hyperuricemia overall (aOR: 1.43, 95% CI 1.21∼1.70), as well as in the age groups of 45∼59 years (aOR: 1.58, 95% CI 1.05∼2.38), 60∼74 years (aOR: 1.54, 95% CI 1.22∼1.94), but not in the age groups of 75∼ years (aOR: 1.33, 95% CI 0.94∼1.89 [*p* = 0.111]). Similar findings were observed in the sensitivity analysis of eGFR as a continuous variable (Figure 4B).

### Additive Interaction Models of Hyperuricemia, Cardiovascular Disease Risk and Age on Chronic Kidney Disease

**FIGURE 5:**
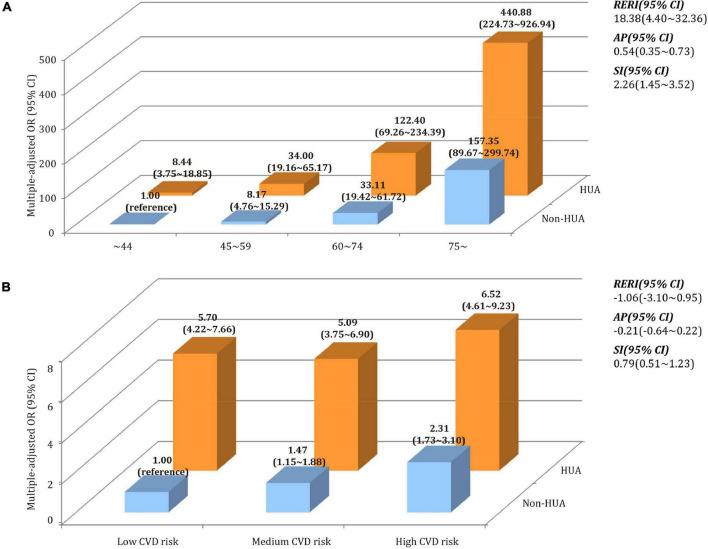
Synergistic effect of the interaction between hyperuricemia, CVD risk, and age on CKD. **(A)** Additive model of hyperuricemia (HUA) + age on CKD. **(B)** Additive model of hyperuricemia (HUA) + CVD risk on CKD.

Figure 5 depicted the additive interaction of hyperuricemia, CVD risk, and age group in relation to CKD. After adjusting for age and gender, participants with hyperuricemia in 75∼ age group had the highest likelihood of CKD when compared to those who aged ∼44 years and without hyperuricemia. The values of RERI, AP, and SI were 18.38 (95% CI 4.40∼32.36), 0.54 (95% CI 0.35∼0.73), and 2.26 (95% CI 1.45∼3.52), respectively. It indicated that coexisting with older age and hyperuricemia, the likelihood of CKD was significantly higher than the sum of the two alone. According to the estimate of AP, 54% of the likelihood of CKD could be attributed to the synergistic effect of *hyperuricemia+age.* However, no such additive interaction between hyperuricemia and CVD risk was not discovered. In a sensitivity analysis using eGFR as a continuous variable, both *hyperuricemia+age* and *hyperuricemia+CVD* risks were found to have additive interactive effects (all *p* < 0.001, Supplementary Figure 1).

## Discussion

In this cross-sectional study, we discovered a negative multiplicative interaction between hyperuricemia and CVD risk, and a positive additive interaction between hyperuricemia and age, both of which have a complex impact on CKD. It was observed that hyperuricemia was more strongly associated with CKD in the low CVD-risk subgroup compared with those in the medium- and high-groups. The significant association between CVD risk and CKD was found only in participants without hyperuricemia. Furthermore, when older age and hyperuricemia coexisted, the combined effect was greater than the sum of their independent effects. To the best of our knowledge, this is the first study in China that identified the age-specific effect modification of metabolic disease and traditional cardiovascular risk on the prevalence of CKD in the general population.

Consistent with previous research (23–25), hyperuricemia was found to be associated with CKD independent of other covariates. Daniel et al. (23) found there was a 7% increase in the risk of CKD for every serum uric acid increase (aOR: 1.07, 95% CI 1.01 to 1.14). Notably, in patients with increased SUA levels, the risk of developing new-onset CKD increased as the duration of follow-up increased, suggesting that hyperuricemia may play an important role in the long-term progression of chronic renal dysfunction (24, 25). Other studies, however, have not found such a positive association. Yasuaki et al. (26) found that, in patients with diabetes, the baseline uric acid levels were not significantly associated with the development of nephropathy. Another cohort study in patients with diabetes found the decline rate of eGFR was not correlated with uric acid (27). To obtain robust results, we established multivariate models by taking CKD and eGFR as outcomes. It was observed that the associations between hyperuricemia and CKD remained significant in all CVD risk groups and were stronger in the low-CVD risk. Furthermore, participants with hyperuricemia had a higher FRS value and lower kidney function since the age group of 18∼29 years. These findings suggest that young participants with hyperuricemia are at risk of early onset of CVD events and kidney dysfunction, despite the absence of clinical hyperuricemia and gout symptoms. Since early-stage of hyperuricemia is often asymptomatic, it has traditionally been overlooked as a simple bystander effect of decreased eGFR (28). Once the kidneys are impaired, urate-lowering treatments are ineffective in slowing the progression of CKD (29). Two negative clinical trials (30, 31), published in the New England Journal of Medicine, suggested that lowering uric acid is unlikely to be beneficial in CKD patients. Moreover, our study found a synergistic effect between age and hyperuricemia on CKD. It is well known that the morphology of the kidney changes with age, and its physiological function continues to deteriorate. Prolonged hyperuricemia exposure to the elderly increases the cumulative risk of kidney damage. The disappearance of the association in the age group of 75∼ years is due to the effect of hyperuricemia on CKD being masked by the complicated comorbidities and smaller sample size.

The specific mechanisms by which hyperuricemia may be associated with CKD have primarily been reported in experimental studies. Firstly, crystallized monosodium urate is suspected of causing urolithiasis, and the subsequent hydronephrosis causes increased intratubular pressure, resulting in decreased renal blood flow and glomerular filtration (32, 33). Secondly, elevated uric acid levels can promote preglomerular arterioles medial thickening and are directly correlated with glomerular capillary pressure (34). Such arteriolar changes in the kidney can result in ischemia and hypoxia, two of the most powerful triggers for tubulointerstitial fibrosis (35). Thirdly, hyperuricemia can hasten renal function deterioration indirectly through hypertension, cyclooxygenase-mediated thromboxane-induced vascular disease, and insulin resistance (36, 37).

Traditional CVD risk factors, such as hypertension, diabetes, and former smoking, were found to be associated with a high prevalence of CKD. The cardiovascular and renal systems have numerous pathophysiological connections. Disease in one organ may cause dysfunction in the other, leading to the failure of both organs (38). On one hand, cardiac dysfunction can increase intrarenal resistance and decrease renal blood perfusion, which further aggravates kidney damage by activating the renin-angiotensin system, increasing inflammatory responses and oxidative stress (39). On the other hand, CKD patients often encountered anemia, sodium-water retention, calcium-phosphorus metabolism disorders and emerging uremic toxin. These pathological changes increase the risk of CVD events and mortality in CKD patients (10). Our data showed that the association between CVD risk on CKD was significant only in participants without hyperuricemia in the stratified analysis. It indicated that hyperuricemia had a stronger link to CKD, which could overshadow the effects of other factors. Previous studies also found that traditional CVD risk factors do not account for the high cardiovascular risk in CKD patients, and that managing CVD in CKD patients was ineffective (5). Therefore, timely intervention in the early stages of hyperuricemia may be more beneficial than in the later stages, when CVD risks have accumulated and kidney damages have occurred.

Our study is unique in that it is a large-scale study of CKD in the Chinese population using standardized sampling, and it uses stratified analyses to evaluate the interactive effects of hyperuricemia, CVD risk and age on CKD. However, some limitations must be mentioned. Firstly, the cross-sectional design does not allow us to examine the effect of hyperuricemia and CVD risk on the progression of CKD. The present associations are not considered causal. Secondly, the diagnosis of CKD was on serum creatinine without taking albuminuria into consideration. It may lead to misclassification for estimating the true prevalence of CKD. Thirdly, selection bias might still exist in this study, although the difference between excluded and included people was mainly due to the exclusion of participants aged below 18 years. Fourthly, high purine diets and concomitant medications (urate-lowering therapy and antihypertensive agents) may affect the estimate of hyperuricemia and CVD risk (40, 41). However, treatment information was not collected for all participants in the CHNS study, which could lead to potential confounding biases.

## Conclusion

Our findings provide evidence that hyperuricemia is associated with CKD independently of CVD risk and age. Interaction analyses revealed that such an association begins at a young age and becomes stronger in the low-CVD risk group, implying a risk of early onset of CKD. Further studies should be conducted to clarify the causal effect modifications and identify novel biomarkers for CKD prediction in various disease contexts. For young adults, early detection of hyperuricemia, routine CVD risk assessment, and timely intervention of modifiable factors are warranted.

## Data Availability Statement

Publicly available datasets were analyzed in this study. This data can be found here: https://www.cpc.unc.edu/projects/china/news.

## Ethics Statement

The studies involving human participants were reviewed and approved by Boards of the University of North Carolina (United States) and the National Institute of Nutrition and Food Safety (China) approved the ethics of CHNS. The patients/participants provided their written informed consent to participate in this study.

## Author Contributions

YL, YF, and XD contributed to the conception and design of the work. YL, BZ, and YX contributed to the acquisition, analysis, and interpretation of data for the work. SJ and WZ participated in data management. YL and BZ drafted the manuscript. XD and YF critically revised the manuscript. All authors gave final approval and agreed to be accountable for all aspects of work ensuring integrity and accuracy.

## Conflict of Interest

The authors declare that the research was conducted in the absence of any commercial or financial relationships that could be construed as a potential conflict of interest.

## Publisher’s Note

All claims expressed in this article are solely those of the authors and do not necessarily represent those of their affiliated organizations, or those of the publisher, the editors and the reviewers. Any product that may be evaluated in this article, or claim that may be made by its manufacturer, is not guaranteed or endorsed by the publisher.

## Abbreviations

AP: attributable proportion due to interaction
BMI: body mass index
BP: blood pressure
CHNS: China health and nutrition survey
CNY: China Yuan
CI: confidence interval
CKD: chronic kidney disease
COPD: chronic obstructive pulmonary disease
CVD: cardiovascular disease
eGFR: estimated glomerular filtration rate
ESRD: end-stage kidney disease
FRS: Framingham risk score
HbA1c: Hemoglobin A1c
HDL-C: high-density lipoprotein cholesterol
IQR: interquartile range
KDIGO: kidney disease: improving global outcomes
MET: metabolic equivalents
RERI: relative excess risk due to interaction
SI: synergy index
SUA: serum uric acid
TC: total cholesterol
WHR: waist to hip circumference ratio
OR: odds ratio.
