# Supplementary material for: Effect Modification of Hyperuricemia, Cardiovascular Risk, and Age on Chronic Kidney Disease in China: A Cross-Sectional Study Based on the China Health and Nutrition Survey Cohort
Source: Front Cardiovasc Med. 2022 Mar 7;9:853917. doi: 10.3389/fcvm.2022.853917 (PMC8934943; doi:10.3389/fcvm.2022.853917)
Supplement: Supplementary file 1 [file Data_Sheet_1.docx]

**Supplementary Table 1.** Comparison of baseline characteristics between the analytic and excluded participants

|  | Total (n=9516) | Analytic participant (n=8243) | Excluded participant (n=1273) | Statistics | p-value |
| --- | --- | --- | --- | --- | --- |
| Age (years) | 48.11±18.20 | 51.59±15.28 | 25.58±19.47 | 54.328 | <0.001^a^ |
| Male (%) | 4520(47.5) | 3790(46.0) | 730(57.3) | 57.129 | <0.001 ^c^ |
| Education (years) |  |  |  | 248.603 | <0.001 ^d^ |
| Never | 1108(11.7) | 1065(12.9) | 43(3.4) |  |  |
| Primary school | 2942(31.0) | 2376(28.9) | 566(45.3) |  |  |
| Junior high school | 3263(34.4) | 2789(33.9) | 474(37.9) |  |  |
| Senior high school | 1054(11.1) | 949(11.5) | 105(8.4) |  |  |
| Post-secondary education | 1110(11.7) | 1048(12.7) | 62(5.0) |  |  |
| Urban (%) | 3095(32.5) | 2730(33.1) | 365(28.7) | 9.935 | <0.001 ^c^ |
| Nationality (Han) | 8393(88.5) | 7274(88.5) | 1119(88.1) | 0.194 | 0.659 ^c^ |
| Total net individual income (CNY) | 11040[5462,19000] | 11080[5578,18894] | 10800[2915,21600] | 1.971 | 0.049 ^b^ |
| Hypertension | 2434(25.8) | 2315(28.1) | 119(10.0) | 179.026 | <0.001 ^c^ |
| Diabetes | 439(4.8) | 414(5.0) | 25(3.0) | 7.198 | 0.007 ^c^ |
| Myocardial infarction | 139(1.6) | 135(1.7) | 4(0.8) | 1.491 | 0.222 ^c^ |
| Smoking status |  |  |  | 36.462 | <0.001 ^c^ |
| Never smoker | 5542(61.0) | 4946(60.0) | 596(70.6) |  |  |
| Former smoker | 1150(12.7) | 1073(13.0) | 77(9.1) |  |  |
| Current smoker | 2395(26.4) | 2224(27.0) | 171(20.3) |  |  |
| Alcohol drinking |  |  |  | 56.838 | <0.001 ^c^ |
| Never drinker | 4521(49.7) | 4011(48.7) | 510(60.4) |  |  |
| Former drinker | 1694(18.6) | 1606(19.5) | 88(10.4) |  |  |
| Current drinker | 2871(31.6) | 2625(31.9) | 246(29.1) |  |  |
| WHR | 0.87±0.08 | 0.87±0.08 | 0.85±0.08 | 11.325 | <0.001 ^a^ |
| BMI (kg/m^2^) | 22.87±3.83 | 23.34±3.48 | 19.81±4.53 | 31.766 | <0.001 ^a^ |
| Systolic BP (mm Hg) | 122.79±19.89 | 124.95±19.21 | 108.85±18.54 | 26.050 | <0.001 ^a^ |
| Diastolic BP (mm Hg) | 79.07±11.69 | 80.20±11.21 | 71.77±12.08 | 23.012 | <0.001 ^a^ |
| FRS | 5.6[2.4,13.2] | 6.3[2.8,13.2] | 1.6[1.2,3.9] | 31.091 | <0.001 ^b^ |
| Uric acid (umol/L) | 308.31±104.31 | 307.80±105.40 | 311.77±96.59 | -1.234 | 0.215 ^a^ |
| HUA (%) | 1523(16.1) | 1321(16.0) | 202(16.6) | 0.222 | 0.637 ^c^ |
| eGFR (mL/min/1.73 m2) | 82.88±21.53 | 78.62±16.89 | 111.64±26.81 | -58.268 | <0.001^a^ |
| CKD (eGFR<60, %) | 1060(11.2) | 1039(12.6) | 21(1.7) | 126.570 | <0.001 ^c^ |

^a^ Student’s t test; ^b^ Mann-Whitney test; ^c^ Pearson test; ^d^ Cochran-mantel-haenszel test

Abbreviation: CNY, China Yuan; WHR, waist to hip circumference ratio; BMI, body mass index; BP, blood pressure; FRS, Framingham risk score; eGFR, estimated glomerular filtration rate; CKD, chronic kidney disease.


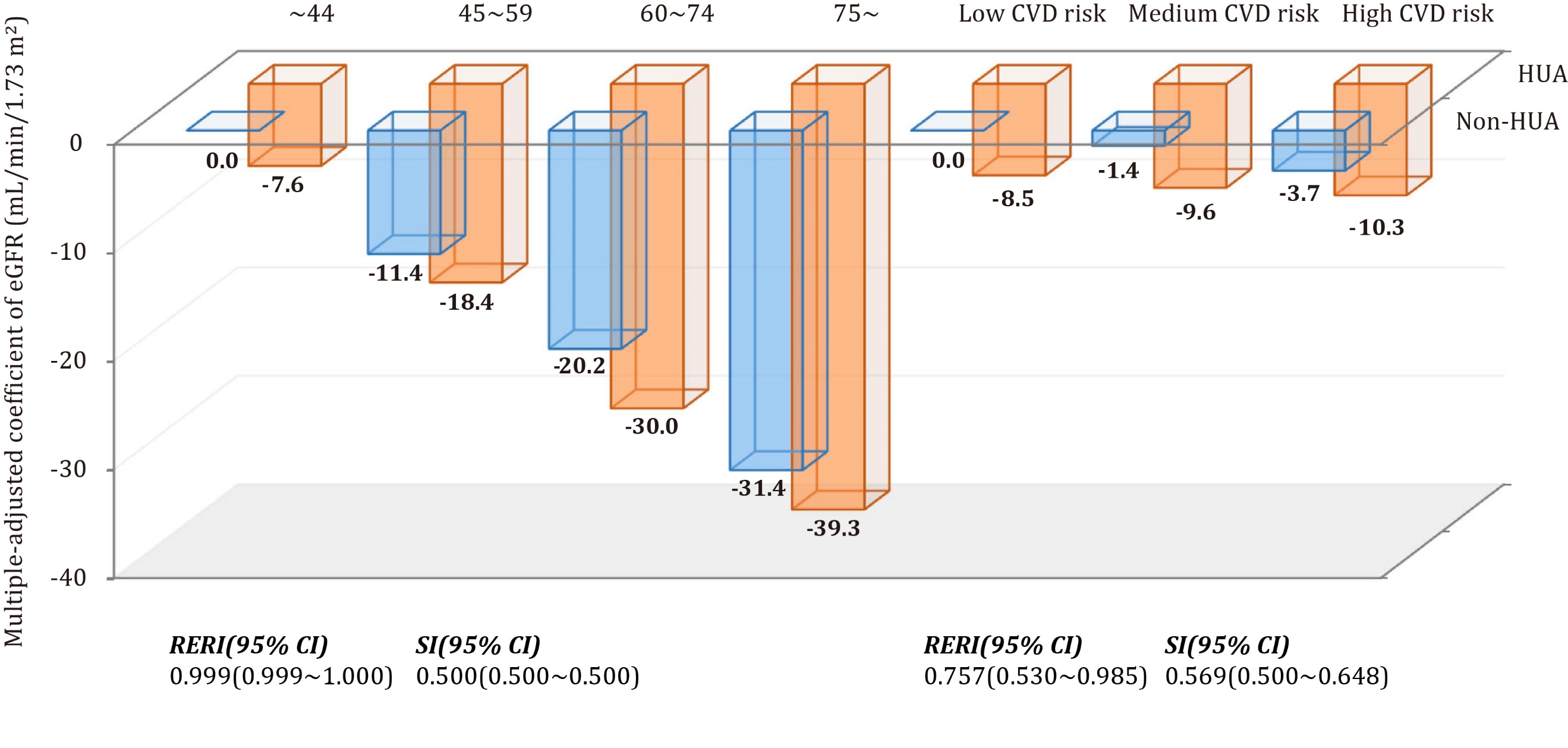
**Supplementary Figure 1.** Synergistic effect of the interaction between hyperuricemia, CVD risk, and age on eGFR

Abbreviation: eGFR: estimated glomerular filtration rate; OR, odds ratio; RERI: relative excess risk due to interaction; SI: synergy index.
